# Determinants of Tobacco Smoking Addiction in Rural Indonesian Communities

**DOI:** 10.1155/2020/7654360

**Published:** 2020-07-17

**Authors:** Jovian Philip Swatan, Sulistiawati Sulistiawati, Azimatul Karimah

**Affiliations:** ^1^Faculty of Medicine, Universitas Airlangga, Surabaya, Indonesia; ^2^Department of Public Health and Preventive Medicine, Faculty of Medicine, Universitas Airlangga, Surabaya, Indonesia; ^3^Department of Psychiatry, Faculty of Medicine, Universitas Airlangga, Surabaya, Indonesia; ^4^Department of Psychiatry, Dr. Soetomo General Academic Hospital, Surabaya, Indonesia

## Abstract

**Purpose:**

To analyze the determinants of tobacco smoking addiction in rural areas.

**Methods:**

A cross-sectional study was conducted on February 2020. The self-administered questionnaire (*α* = 0.908) and Perceived Stress Scale–10 were used as tobacco smoking determinants and the WHO ASSIST questionnaire V3.0 to determine its addiction risk. Their correlations were analyzed by Spearman's rank-order approach using the SPSS version 23.0.

**Results:**

Among 75 male respondents that participated in this study, those on low, moderate, and high addiction risk were 45 (60.00%), 23 (30.67%), and 7 (9.33%), respectively, and significantly correlated with the research questionnaire that consisted three parts: 1. awareness toward the health risk; 2. social control; 3. mass media role in tobacco smoking (*p*=0.014, 0.004, and 0.009 respectively), but there was no significant correlation with the stress level (*p*=0.287).

**Conclusion:**

Increased awareness toward the health risk, good social control, and mass media reporting the danger of tobacco smoking is significantly in correlation with the decreased addiction in rural areas. However, the high perceived stress has no correlation with its increase.

## 1. Introduction

Indonesia is the world's 4^th^ most populous country, where 43.3% of its population lives in rural areas, and smoking remains as a health problem in this nation [1]. According to the 2018 Basic Health Research (Riskesdas), tobacco prevalence was predominant among the age of 15 years and above (33.8%), and majority are male smokers [2]. A study among the U.S. adults shows that it is more common in their rural areas compared with that in the urban settlements [3]. Similar findings in Indonesia also recorded the same result (36.8% vs 31.9%) [4].

Tobacco addiction is an Indonesian public health issue, with a steady rising of the incidence and also the increasing mortality rate because cardiovascular diseases are associated with the high prevalence addiction of tobacco smoking. [5]. Aditama observed that most of the male participants were heavy smokers with an average of 7.6 cigarettes per day [6], while a different study showed 21.4% [7].

Several studies have outlined the causes of smoking behaviour such as lack of knowledge, socioeconomic factors, information through media, and stress or negative life-related events [8–11]. However, majority of those studies were conducted in urban areas. Therefore, this study aims to analyze the determinants of tobacco smoking addiction in rural areas.

## 2. Methods

This was a cross-sectional study conducted on February 2020 at Songgon district, Banyuwangi Residence, East Java. It lies among the border of Bondowoso and Jember Residence, which are dominated by Madurese, Javanese, and Osing (Banyuwangi natives) ethnicities. These represent those of the rural East Java, which is the 2^nd^ province with the highest number of populations in Indonesia [1]. The respondents were male local villagers aged 15 year old and above. It was conducted under the Community Medicine education training program and organized by the Faculty of Medicine, Universitas Airlangga, on a one-week period. Using sample size calculation, the minimum requirement was 75 responses [12]. Consecutive samplings were carried out until the minimum number is fulfilled. The authors had provided standardized training programs for all interviewers prior to the survey administration.

The respondents were requested to complete three questionnaires, consisting of tobacco addiction determinants, Perceived Stress Scale–10 (PSS-10), and WHO ASSIST v3.0 questionnaire for tobacco. All were given in Indonesian language. The first question list consisted of three sections as follows: (1) health risk awareness, (2) social control, and (3) mass media role in tobacco smoking. With each statement, the respondent's response were placed on a 4-point Likert Scale, where 1 and 4 indicated strongly “disagree” and “agree,” respectively. Each section had a maximum score of 60 points for the first section and 40 points for the second and third sections. This questionnaire has been prevalidated and tested for reliability with alpha = 0.908.

The Indonesian version of the PSS-10 was adapted from the previous studies with *r* = 0.632 and alpha = 0.857 [13], while the WHO ASSIST v3.0 questionnaire was from the Indonesian Ministry of Health's booklet.

### 2.1. Statistical Analysis

The acquired data were analyzed using IBM SPSS Statistics for Windows ver. 23.0 (IBM Corp, Armonk, USA), which were expressed as mean ± standard deviation. Correlation between demographic and addiction risk was measured using both the Spearman's rank-order and Fisher's exact test, while those between the scores obtained from tobacco addiction determinant questionnaire and PSS-10, based on WHO ASSIST v3.0 questionnaire, were measured using Spearman's rank-order only. A *p* value of <0.05 was considered as statistically significant.

### 2.2. Ethical Clearance

This study followed the principles of Helsinki's Declaration and also received the permission from the Faculty of Medicine, Universitas Airlangga, before it began (ethical clearance no. 52/EC/KEPK/FKUA/2020). All respondents had presented their signed consents prior to their inclusion in the study. Details that might disclose the identity of the respondents were omitted.

## 3. Results

### 3.1. Demographic Data

A total of 75 responses were collected and validated. The mean age of the respondents was 44.04 ± 13.10. Most of them worked as a self-employee/subsistence. Regarding the education level, most respondents were of senior high school levels or higher. It was also observed that most of them were married and living with 4–6 persons in their homes. The demographic data were presented in Table 1.

### 3.2. Determinants of Tobacco Smoking Behavior

In the first section, respondents were asked about their views on a tobacco smoker, the introducer, the place, and its health effects. A higher score showed that they were more aware of the health risks of tobacco smoking. The mean point obtained was 42.93 ± 7.03, while 58 and 27 were the maximum and minimum achieved scores, respectively.

In the second section, the respondents' opinions were enquired based on smoking in social settings. A higher score signifies that they could maintain good social control regarding tobacco smoking. The mean point was 29.53 ± 4.9, while 40 and 15 were the highest and lowest scores, respectively.

While the third sections were enquired about the mass media role against smoking behaviour. A higher score means that they were more aware of the media reporting the dangers of tobacco smoking. The mean point recorded was 30.20 ± 4.71, while the maximum and minimum obtained scores were 40 and 15, respectively.

The PSS-10 is a questionnaire used to measure the perception of stress. A higher score indicates that the respondent is having a high level of stress. Smoking was considered one of the behavioural responses during stressful conditions. The mean score acquired was 14.96 ± 5.67, while the highest and lowest acquired points 26 and 0, respectively.

### 3.3. Addiction Risk and Demographic Data

The WHO ASSIST v3.0 is used to measure the addiction risk toward smoking. The scores were categorized into three, namely, low (0–3), moderate (4–26), and high (more than 26), and the number of respondents on each class was recorded as 45 (60.00%), 23 (30.67%), and 7 (9.33%), respectively.

Table 1 describes the correlation between demographical data and tobacco addiction danger. It was observed that the risk did not significantly correlate with age (*p*=0.241), occupation (*p*=0.553), education level (*p*=0.940), marital status (*p*=0.593), and number of persons in each home (*p*=0.873).

### 3.4. Addiction Risk and Determinants of Smoking Behavior

The scores of each questionnaire sections and PSS-10 were compared based on the respondents' addiction risk (Table 2). We observed that those in the lower group scored the highest point in all the three sections (44.62 ± 7.48, 30.93 ± 4.91, and 31.33 ± 4.72, respectively). Respondents in the moderate group got the lowest on the first section with an average of 40.30 ± 5.49 scores, while those on the high category got the least in the second and third sections (26.43 ± 5.35 and 28.43 ± 3.87 points, respectively). Spearman's rank showed an inverse correlation between score achieved on the questionnaire and addiction risk (*r* = −0.283, −0.328, and −0.301, respectively) and those in all the three sections (*p*=0.014, 0.004, and 0.009, respectively). On the PSS-10, those in the lower group had a low score compared with those in the moderate and higher groups (14.20 ± 6.31 vs 15.48 ± 4.73 and 17.57 ± 2.94). However, this study shows that there is no significant relationship between the PSS-10 score and the risk of tobacco addiction (*p*=0.287).

## 4. Discussion

Currently, only one study that had been conducted to analyze the determinants of tobacco smoking addiction in rural areas of Indonesia [14]. Previous studies show that smoking is more prevalent among men than women in rural areas [2,15]. The preliminary survey also found out that no female smoker was recorded in Songgon district; therefore, only male respondents were recruited to participate.

A better awareness of tobacco health dangers is associated with a lower addiction risk and a desire to quit smoking [16,17]. This is due to the high awareness level of the dangers which sabotages the victims' experience, making it a worse exposure, and as a result, indirectly reducing the risk of addiction [18].

Several studies reported that social environments and stigmatization from smoking behaviours have led to decreased smoking rates [9,19]. Maintaining a good social control means that a person is capable of inhibiting smoking while maintaining an ethical interaction with others. In Indonesia, especially in rural areas, a cigarette is usually offered during social meetings and occasionally as a sign of friendship. Another cultural aspect is the politeness rejection of a host's offer, which is considered impolite and may offend them [6]. Therefore, the smoking rate is unsurprisingly high, but its addiction is a different matter as it is multifactorial and often depends on personal experiences. The authors assumed that social meetings contributed to smoking addiction for those that socialize (extraverted people). However, further study is needed to investigate this assumption. It was presumed that social control might be a better determinant of the addiction risk.

The mass and social media campaigns were thought to be effective in changing smoking behaviour among adults [20,21]. In contrast, it was also assumed that smoking advertisements have been enhancing their habits toward tobacco [8]. In Indonesia, campaigns to stop smoking or sensitization on its dangers is rarely carried out, but tobacco advertisement is always seen everywhere [22,23]. Again, it must be emphasized that tobacco addiction is multifactorial and depend on the respondents' perception. It is argued that a person's awareness of mass media reporting the danger of smoking may be a better determinant.

This research made some findings different from those of the previous studies that the stress level were not significantly related to addiction risk [24,25], since the stress level in rural areas is lower than that in the urban settlements. It was also observed that the urban settlements experienced more distress compared with the rural areas. [26]. Besides environmental and socioeconomic aspects, rural areas tend to support each other through *gotong royong*, a term which encompasses communal service and mutual assistance without hesitancy to those in need [27]. Therefore, it is assumed that the lower stress levels may lead to a stress-related smoking behaviour, which has not been considered in this research.

This study has several limitations. Firstly, the limited time which restricted the sample size and method. However, the authors managed to reach the minimum sample required. Secondly, using only a single district may not represent rural areas in other provinces or islands in Indonesia. Thirdly, the authors did not study the smoking behaviour and the social background that lead to addiction. Therefore, further study is recommended with more sample sizes in several other rural areas.

## 5. Conclusion

Tobacco smoking remains a nationwide problem in Indonesia. Results show that high perceived stress has no correlation with the increased addiction risk in rural areas. However, the increased awareness of its health dangers, good social control, and mass media campaign is significantly in relationship with its decrease. Therefore, this study could be a recommendation of the smoking prevention program to address more of the issues rather than focusing on stress management for the population.

## Figures and Tables

**Table 1:** Demographic data of respondents and correlation to tobacco addiction risk.

Demographic data (*n* = 75)	*N* (%)	*p*
Age		
Range	18–70	0.241a
Mean ± SD	44.04 ± 13.10	

Occupation		
Self-employee/subsistence	28 (37.33)	0.553b
Civil servant/nongovernment employee	24 (32.00)	
Farming	17 (22.67)	
Student	3 (4.00)	
Unemployed/retired	3 (4.00)	

Education level		
Completed elementary school	17 (22.67)	0.940a
Completed junior high school	14 (18.67)	
Completed senior high school or higher	44 (58.67)	

Marital status		
Married	65 (86.67)	0.593b
Not married	10 (13.33)	

Number of person in home		
1–3	28 (37.33)	0.873a
4–6	46 (61.33)	
More than 6	1 (1.33)	

^a^Using Spearman's rank-order; ^b^Using Fisher's exact test; *p* < 0.05 is considered significant.

**Table 2:** Comparison of mean questionnaire score and addiction risk to tobacco.

Questionnaire	Addiction risk			*r*	Sig.
	Low (*n* = 45)	Moderate (*n* = 23)	High (*n* = 7)		
Section 1	44.62 ± 7.48	40.30 ± 5.49	40.71 ± 6.02	−0.283	0.014^*∗*^
Section 2	30.93 ± 4.91	27.74 ± 3.83	26.43 ± 5.35	−0.328	0.004^*∗*^
Section 3	31.33 ± 4.72	28.52 ± 4.39	28.43 ± 3.87	−0.301	0.009^*∗*^
PSS–10	14.20 ± 6.31	15.48 ± 4.73	17.57 ± 2.94	0.125	0.287

^*∗*^ *p* < 0.05 is considered significant.

## Data Availability

The data used to support the findings of this study are available from the corresponding author upon request.
